# Home care workers’ experiences of work conditions related to their occupational health: a qualitative study

**DOI:** 10.1186/s12913-021-06941-z

**Published:** 2021-09-14

**Authors:** Sunniva Grønoset Grasmo, Ingeborg Frostad Liaset, Skender Elez Redzovic

**Affiliations:** Faculty of Medicine and Health Sciences, Department of Neuromedicine and Movement Science, Norwegian University of Science and Technology (NTNU), Tungasletta 2, N-7047 Trondheim, Norway

**Keywords:** Work conditions, Occupational health, Home care workers, Home care services, Qualitative study

## Abstract

**Background:**

The need for home care workers (HCWs) is rapidly growing in Norway due to the increasingly growing elderly population. HCWs are exposed to a number of occupational hazards and physically demanding work tasks. Musculoskeletal disorders, stress, exhaustion, high sick leave rates and a high probability of being granted a disability pension are common challenges. This qualitative study explored the views of HCWs on how working conditions affect their safety, health, and wellbeing.

**Methods:**

A descriptive and explorative design was utilised using semi-structured individual interviews with eight HCWs from three home care units in a middle-sized Norwegian city. Interviews were conducted in the Norwegian language, audio-recorded, and transcribed verbatim. The data was analysed by systematic text condensation. Key data quotes were translated into English by the authors.

**Results:**

HCWs reported that meaningful work-related interactions and relationships contributed to their improved wellbeing. Challenging interactions, such as verbal violence by consumers, were deemed stressful. The unpredictable work conditions HCWs encounter in users’ homes contributed to their exposure to environmental hazards and unhealthy physical workloads. This was the case, although the employer promoted ergonomic work practices such as ergonomic body mechanics when mobilising and handling of clients, using safe patient handling equipment. HCWs perceived high level of individual responsibility for complying with company safety policies and practices, representing a health barrier for some. Organisational frameworks created unhealthy work conditions by shift work, time pressure and staffing challenges. Performing tasks in accordance with HCWs professional skills and identity was perceived as health-promoting.

**Conclusions:**

This study suggests that unpredictable working conditions at users’ home can adversely affect the safety, health, and wellbeing of HCWs. The interaction between the unpredictable environment at users’ homes, HCWs’ perceived high level of individual responsibility for complying with company safety policies and practices, and staffing challenges due to sickness-related absences upon the workplace creates tense work conditions with a negative influence on HCWs health.

## Introduction

Home care workers (HCWs) provide social- and healthcare to all citizens in Norway regardless of age, gender, financial situation, social status, family situation, or ethnic background. They provide services to users discharged from the hospital, the elderly, users with disabilities, chronic diseases, drug addiction issues, mental health challenges, and terminally ill patients [1, 2]. Their services are delivered at the users` home and include medical assistance and nursing care, practical assistance to maintain the activities of daily life, rehabilitation, as well as end-of-life care [1]. The term user is widely used because home care services in Norway are based on the universal welfare model implying that all citizens can get the help they are eligible for free, and there is a high focus on their empowerment.

In the early 1960s, the Norwegian public home care system emerged as a part of an elderly care policy aimed at avoiding segregation, social exclusion, and passivity among the elderly. Due to decentralising reforms that came into effect during the mid-1980s and early 1990s, local authorities were assigned the responsibility of providing a range of statutory services. As a consequence, home care services became much more comprehensive [1, 2]. Approximately 196,000 users received services at their homes during 2019 [3], an increase of 13% from 2015. The services increased the most (16%) for the group under the age of 67.

HCWs are registered health professionals with higher university education (e.g., nurses, occupational therapists, physical therapists) or have certifications for home health aides. Some of them work as assistants who are trained at the workplace to assist users [1]. Approximately 146,000 employees worked at home care services in Norway in 2020 [3].

Salary surveys for 2019–2020 showed that HCWs certified as home health aides earn between 44,000–50,000 USD in a year (37,5 work hours per week) [4], while nurses [5] and occupational therapists [6] earn between 55,000–65,000 USD in a year (37,5 work hours per week) depending on age, seniority or position type. Statistics showed an increase in the number of immigrants who work in the care services in Norway. They are from more than 160 different countries, where people with a background from EU countries in Eastern Europe and Asia are a large group. The proportion of immigrants measured on agreed man-years in municipal care services varies significantly between different parts of the country [7].

Studies have shown that Norwegian HCWs report high psychosocial work exposures (e.g., workload pressure, time pressure), high levels of strain, exposure to various occupational hazards and physically demanding work tasks. Musculoskeletal disorders, overexertion injuries, chronic stress, and exhaustion are prevalent health problems among HCWs [8–12]. Extensive efforts have been made to improve working conditions for HCWs in Norway, but the impact of such efforts in terms of improved health is demonstrably limited [1, 13–15]. Sick leave rates, probability of being granted a disability pension and turnover remain high in the Norwegian home care services [1, 13–15]. Attracting and retaining HCWs is challenging [1]. Although the situation underlines the need for insight on how HCWs perceive the influence of their work upon their health, only a few qualitative studies have been conducted on the occupational health of HCWs in Norway [13, 14].

Andersen and Westgaard [13] found that rationalisation measures such as new work programs and technology, restructuring, unit mergers and management replacement were perceived by HCWs as major sources of stress, having harmful and interruptive effects on the work environment intervention aimed to promote their working conditions. HCWs were often impaired by work-related stresses and worries, wear and tear injuries, exhaustion, neck and shoulders tension, headaches, back pain, and various strain injuries. Moreover, their findings showed that high sick leave was a twofold problem for the service providers. Sick leave was regarded as a symptom of a strenuous work situation created by substantial pressure and organisational changes. However, it was also a source of additional stress for the remaining workers because they became further overloaded with work. Another qualitative study reported that employees at different organisational levels in the home care services expressed different understanding of factors related to the working conditions of HCWs, such as time pressure, work tasks, work program, organisational changes, budget model, budget allocation and coping strategies [15].

More studies are needed to identify a full range of working conditions that affect the safety, health, and wellbeing of HCWs. As a consequence of the demographic trends in the coming decades and rationalisation measures conducted in the field to increase operating efficiency and service quality, [2, 13, 16, 17] HCWs are sure to face even more demanding and stressful work conditions in the future [18, 19]. Furthermore, as the elderly population increases and the proportion of working-age people shrinks [20], the need for assistance from qualified home health care professionals in the Norwegian population will continue to grow without corresponding access to labour and skills [21]. The present study aims to explore the views of HCWs on how working conditions affect their safety, health, and wellbeing.

## Methods

In the present study, a hermeneutic-phenomenological approach was utilised for in-depth exploration of HCWs experiences on their occupational health in the context of their conditions at work [22, 23]. The study was designed to answer the research question by allowing the interactions between the interviewer and participants to shape the data collection and analysis (inductive approach). The qualitative study consisted of individual, in-depth, semi-structured interviews with HCWs, and adheres to the Consolidated Criteria for Reporting Qualitative Research (COREQ) guidelines to achieve comprehensive and explicit reporting of the study [24].

### Research team and reflexivity

Team reflexivity was consciously and actively utilised to ensure productive teamwork as well as rigour for the research [25]. The research team consisted of three researchers (SGG, IFL, and SR as senior researchers), and was not involved in the home care services and did not know the participants prior to study inclusion. None had experience with home care services. SR and IFL had established a relationship with the home care units where the study was conducted due to collaboration on a research project. During the research process, team members shared their reflections, confronted inconsistencies in their interpretations, and continuously considered alternative evidence in their discussion.

### Selection of participants

Employees from three home care service units in a medium-sized city in Norway were invited to participate. HCWs were registered health professionals with university education (e.g., nurses, occupational therapists), certified home health aides and assistants.

Inclusion criteria for the study were (a) employment in the home care services for at least six months, (b) holds training as assistant/certification as a home health aide/bachelor’s degree in health and social sciences, and (c) holds a minimum of 50% full-time equivalent (FTE) position. The recruitment process began via a collaboration with the heads of the home care units. The leaders informed the HCWs about the study, orally and by distributing written information, and those willing to participate contacted the researcher by mail or telephone. Out of 99 employees, eight employees contacted the researcher and took part in the study. Further recruitment was not conducted as the variability of the HCW population sample was deemed satisfactory, and data saturation was reached at an acceptable level to meet the objective of this exploratory study. The saturation point was identified based on fewer surprises and no more emergent patterns in the data. Thus, the ability to obtain additional new information was attained. Participant characteristics are summarised in Table 1*.*

**Table 1:** Characteristics of the participants

Characteristics	*n* = 8
**Gender**	
Male	4
Female	4
**Age (year)**	
20–25 years	3
25–30 years	1
30–35 years	4
Mean	27,62
Median	28,53
**Ethnicity**	
Norwegian	8
**Qualifications**	
Bachelor degree in nursing	4
Bachelor degree in occupational therapy	1
Certification as home health aides	3
**Clinical work experience in-home care services (year)**	
6 months - 1 year	1
2–4 years	3
7–10 years	4
**Percentage of employment**	
80 percentage	1
100 percentage	7
**Home care units**	
Unit 1	2
Unit 2	2
Unit 3	4

### Data collection

The first author conducted semi-structured in-depth individual interviews over two weeks during February 2020 at work-based locations and times convenient for each of the eight participants. Data collection was not affected by the COVID-19 pandemic. The researcher made reflexive notes throughout the data collection process and immediately after each session. The interviews lasted between 50 and 90 min.

A flexible semi-structured interview guide was designed based on literature findings [26] and utilised to facilitate exploring the experiences and understandings of each participant. The interview guide consisted of a comprehensive set of open-ended questions and other additional relevant prompts (e.g., *‘How do you perceive your work conditions?’; ‘What does a typical workday look like for you?’; ʻHow does your experience of working conditions influence your health?ʼ; ‘How do your experience work conditions influence your health?’; ‘Can you describe how you cope with challenges in your workday and how it affects your health?’)*. To create rich descriptions, the main questions were followed up with probe questions such as *‘Can you give an example of this?’, ‘Can you elaborate on this?’*

To ensure a collection of participants` subjective interpretations, the researcher summarised descriptions with follow-up questions during the interview and performed a debriefing at the end of each interview.

### Data analysis

All interviews were audio-recorded with permission and transcribed verbatim. Systematic text condensation in four steps where utilised, a method inspired by and based on Giorgi’s psychological phenomenological analysis [27]. In the first step, we established an overview of the raw data. We read the 137 pages (average of 510 words per page) of the transcript several times and notated them with initial thoughts to establish a total impression of the whole and get an idea of the main themes associated with participants’ experiences of work conditions and their perceived health. After the sense of the whole statement had been grasped, the researchers` attention was attracted by preliminary themes such as *relations, demanding work tasks and organisational framework.*

In the second step of the analysis, we systematised and identified data elements that may illuminate the study question. The first author systematically reviewed the transcript line by line to identify meaning units, a text fragment containing some information about the research question. For instance, *muscular pain, performing extra work tasks and transferring users.* The emergence of meaning units continued until topic saturation. At that point, no new themes emerged. Then the researcher started coding by identifying, classifying and sorting meaning units potentially related to code groups, for instance, *health risks and health-promoting.* Parts of the text were decontextualised and temporarily removed from their original context for cross-case synthesis, with the main themes in mind. At this stage, commonalities and differences within and across the coding groups were examined.

In the third step of the analysis process, the data material no longer appeared as 137 pages of the transcript. It was reduced and organised to a few codes’ groups containing units of meaning that demonstrate a diversity of nuances describing different aspects of the meaning and a capacity to reveal aspects of the connection between HCWs experiences of work conditions and their perceived health. The understanding of the initial preliminary themes from the first step was revisited and evaluated. With a focus on the study question and bearing in mind not to let preconceptions from the preliminary themes of the first step influence the process, the researcher sorted the meaning units of the code groups into sub-categories, for instance, *relationships with users* and *individual responsibility.* By reviewing the sub-categories within the same code group, several aspects representing the thematic content of the code group were created. The first author checked the validity by referring back to the original transcript and making sure the connections were evident. The same producer followed for each of the remaining code groups.

In the fourth step of the analysis, the researcher synthesised all of the transformed meaning units into a consistent statement regarding the participants’ experiences. A table of the main categories and an introduction to each sub-category and meaning units was created (see Table 2)*.* In all of the four steps of the analysis, the researcher aspired to build a bridge between the raw data and the results while still ensuring that the synthesised results would reflect the wholeness of the original material and provide the best possible picture of the participants’ views. The quotes presented in this article are from the individual interviews and are used to illustrate the categories of the data. The researcher translated and edited the Norwegian citations into a more readable form by removing pauses and redundant words.

**Table 2:** Overview of the analysis process and the result

Main Categories (Step 4)	Sub-categories (Step 3)	Meaning units related to code groups (Step 2)	Preliminary themes (Step 1)
Work-related relationships	***Relationships with colleagues*** ***Relationships with users***	*Health promoting:* Support and positive feedback, Social fellowship, Professional development, Constructive conversations. *Health promoting:* Close bonds to users, Meaningful and personal reward to make a positive difference in users’ lives, Being appreciated. *Health risks:* Grief when users became sick or died, Verbal violence, Sexual harassment, Stigmatization (care profession is for women).	Relations
An unpredictable workplace	***Environmental hazards*** ***Unhealthy physical workloads*** **The perceived responsibility for complying to company safety policies and practices**	*Health risks:* Challenging driving and travelling conditions, Ice and snow in the winter, slippery weather, as slip, trip and fall hazards, Unhygienic Environment, Headaches and nausea, Unhealthy air quality and bad smells, Dust and dirt, Lack of pet related hygiene, Clutter, garbage and hoarding, Tobacco Smoke, Exposed to second-hand smoke, Smell from cigarette smoke steeps into clothing. *Health risks:* Users were not willing to make adaptions for use of equipment at home, Limited workspaces, Small and tight rooms, particularly the bathroom, kitchen, and bedroom, Difficult to use appropriate equipment in users home and maintain ergonomic principles, Awkward work postures, Pain in the back, Challenges to maintain ergonomic guidelines because of height and body size, Strenuous work postures on the neck, back, and shoulders, Stiff and pain in the musculature, Pressure to be effective and perform tasks quickly, Multitasking and rushing create unhealthy body positions and incorrect lifting techniques, Unexpected moves, trips or falls from users create unhealthy body positions and incorrect lifting techniques *Health promoting:* Some are careful and set stricter limits to protect themselves. *Health risks:* Responsible for engaging in the appraisal and facilitation of a healthy workplace, Challenges due to different perceptions of working conditions, Don’t dare to ask for help, Push their own limits in order to help users.	Uncertainty Demanding work tasks Hazards Musculature pain Working alone Stress
Organisational conditions	***Shift work, time pressure and staffing challenges*** ***Facilitating professional identity***	*Health risks:* Shift work (Disturbed sleep patterns, Reduced sleep quality and poorly rested), Time pressure **(**Increasing demands and more efficiently, Skipping lunch, Not going to the toilet, Dizziness, nausea, blood pressure problems, seizures and palpitations, Headaches, tension and strain in the neck and shoulders. Mental and emotional strain**,** Exhaustion and burnout, Bad feelings for too little time to meet users psychological and social needs), Staffing challenges (Often as a consequence of HCWs on sick leave. Creates more pressure and stress on the remaining workers who perform extra tasks and take on extra responsibilities). *Health promoting:* Appropriate tasks to their professional competence and identity, Able to use professional competence, Opportunities for special training and further education.	Hectic workdays Organisational framework Being professional

Trustworthiness was ensured by investigator triangulation in the analysis to obtain optimal inter-subjectivity [28]. Furthermore, we used the criteria of credibility, dependability and transferability to ensure the quality of the research [29]. All three authors were active in the discussion and decision-making through all steps of the present study, while the first author was responsible for the practical implementation. The first author conducted all interviews to provide consistency and ensure the credibility of the data collection. While all three authors read interview transcripts, data coding was first performed by the first author, and the coding was then negotiated in an analysis discussion involving all authors until a common understanding was reached. In addition, all three authors discussed the findings and interpretations of the subgroups and main categories until agreement was reached. This process is referred to by Lincoln & Guba [29] as peer checking, which is a substitute for interviewees’ checking of transcripts and is a valid means to increase the credibility of findings. The study’s dependability was addressed using the same interview guide for all interviews, audiotaping, and transcribing the interviews verbatim. Transparency was achieved by providing the reader with a display of the data analysis steps (Table 2). The transferability of our findings to another context was enhanced by using illustrative quotations from the data. Drafting and revision of the manuscript was also conducted in collaboration to ensure scientific dessimination.

### Ethics

This study was approved by the NSD-Norwegian Centre for Research Data (ref.no. 424313). All participants willingly volunteered to participate in the study and to share their views. They received written information about the project when asked to participate, including information about the right to request access to and to delete and/or correct their own personal data and the right of each participant to withdraw from the study at any time without consequences. Participants gave written informed consent on the day of the interview. The interviewer made them aware that they did not have to answer any questions that made them uncomfortable.

## Results

The interviewed HCWs usually worked alone with users of all ages and backgrounds and walked, cycled or drove a car to users’ homes. They worked shift work five days a week (37,5 work hours), described as employment practice designed to provide service across the morning and evening each day of the week. The user group typically consisted of elderly users with mental health and/or addiction challenges, disabilities and/or chronic diseases, as well as the terminally ill. The informants also delivered services to users discharged from the hospital.

All HCWs reported that they provided basic healthcare, including assisting users in maintaining hygiene, distributing and delivering medicine, performing catheterisation and ostomy procedures, dressing and taking on and off compression stockings, cooking, feeding, and cleaning. They also lifted, transferred, and repositioned users. All HCWs had administrative responsibilities. They worked as medical administrators, created work schedules, and handled other paper and telephone-based work.

The HCWs described the primary difference between their roles. When it comes to the home care nurses’ role in the users’ lives, they provide more advanced medical care and specialised medical procedures such as wound care, blood tests, and the administration of injections in addition to basic health care. Due to liability and legality, workers certified as home health aides or occupational therapists cannot perform specific medical tasks. However, home health aides reported performing some medical procedures under the supervision of a nurse. Dissimilar to the nurses and home health aides, the occupational therapists also had a coordinator position in reablement located in the municipality. The informant mapped users’ functional levels and their assistance needs and made recommendations for assistive technology that can improve users’ quality of life.

Three main categories were identified when HCWs elaborated on their occupational health related to their work conditions: *‘Work-related relationships’*, *´An unpredictable workplace´,* and *‘Organisational conditions’.* A detailed overview of the three main categories, subcategories and meaning units is presented in Table 2.

### Work-related relationships

Relationships with colleagues provided social fellowship, support and positive feedback, constructive conversations and professional development. They were perceived as a health-promoting factor and a reason to remain at the job: ‘*The social relationship with colleagues is one of the best parts about the job …*. *We laugh and have fun at work …. It makes me happy’.*

Meaningful relationships to users were also experienced as a positive factor for their occupational health: *‘Sometimes the users may feel like family because we become so close …*. , *it does not always feel like you are at work when visiting them’.* It was perceived as meaningful to be appreciated and make a positive difference in users lives.

On the other hand, participants occasionally experienced their relationships with users as having a negative impact on their health. Emotional strain and feelings of grief were reported as negative outcomes that could arise from becoming too emotionally attached to users or their families, especially when users became sick or died: ‘*It’s absolutely awful when users have died … and I took it pretty heavy really … I struggled a lot because of it’.*

Furthermore, experiences of being exposed to verbal violence when visiting users`.

homes were commonly described: *‘When users stand and shout ugly things to me … I’ll be like: “No, I can’t stand and listen to this”’.* Users’ unpleasant behaviour was, according to participants, often a consequence of their health condition. Moreover, female participants described experiences of uncomfortable verbal and physical and/or sexual behaviour from male users: *‘ … There was a user who held me tight and tried to kiss me … it was last year … ’.* Male participants on the other side reported being stigmatised by elderly users who believe the home care profession is for women: *‘Not all users like male nurses … they want visits, from a woman and then...and I had thoughts about quitting my job, because I got so tired of the stigmatising’.*

### An unpredictable workplace

The users’ homes as workplaces were described as personable, homely, less organised and less controlled, and more unpredictable than care environments in institutional settings. When reflecting on having people’s private homes for their workplaces, several participants emphasised the importance of being able to adapt to different surroundings. According to participants, being humble and respecting the users, their boundaries, and how they want to live their lives was crucial to getting along with users in their homes. The surroundings in users’ homes were described as expressions of the users` themselves and reflections of their personalities. These unpredictable environments could potentially expose them to (i) different environmental hazards, (ii) unhealthy physical workloads, and (iii) the individualisation of the responsibility for engaging in the appraisal and facilitation of healthy working conditions.

#### Environmental hazards

When visiting users’ homes, the participants experienced several environmental hazards with the potential to endanger their occupational health. Driving was reported as a workday stressor that could create dangerous situations. Ice and slippery streets in the winter were described as slip and fall hazards when transferring between homes: *‘I stress when I drive … and I think I drive faster than I should … because that’s where I’m going to save time‘.*

Unhygienic work conditions inside some users` homes were commonly reported: *‘It was so dirty and messy inside the home... and I remember sitting on my knees on the floor, on a plastic bag, because it was so dirty there’.*

Further, the participants reported being exposed to second-hand smoke and experiences of adverse health consequences when visiting the homes of users who smoked: *‘We have rights to a non-smoking working environment … but our workplace is in the home of people, and they are allowed to smoke there if they want’.*

#### Unhealthy physical workloads

The participants reported high degree of support from mangers and the leadership to ensure their safety, health and wellbeing: ʻ*I experience my employer as very supportive and promotes our health and safety … but also … work-life balance … Last year, i felt chronically exhausted and frustrated at work …. Ehhm … I kept making small mistakes all the time and … I was stuck in a cycle of unproductiveness... And my employer, he told me I needed to make a doctor’s appointment … He saw that I was burned out and needed a break ..ʼ.* The leadership and managers were also engaged in promoting ergonomic work practices such as ergonomic body mechanics when mobilising and handling of clients, using safe patient handling equipment.

Despite high focus on ergonomics at the organisational level, greater availability of ergonomics assessments and collaboration among colleagues and the teamwork involved in physically challenging work tasks, participants still reported experiences of unfortunate physical strain and unhealthy body movements and positions, experiences attributable to working conditions in various homes: *‘We had a user where we performed wound care two hours every day, over several years.... The user had his foot on a chair, and we sat with our back bent forwards for two hours … and over several years! ...no, it’s not healthy’.*

Consequently, participants reported stiff and tense muscles, muscular pain, and injuries, particularly the neck, shoulders, feet, and back. Inflammation in the hands and fingers caused by performing monotonous tasks was also a major problem. The need for treatment by a physiotherapist and/or sick leave was reported as common consequences of these health problems.

Tight schedules and unexpected movements from users where the participants had to react quickly resulted in unhealthy body positions and incorrect lifting techniques: *‘There is always so much going on and a lot to do...I don’t always think about if I’m doing this right or not for my body’.* Further, not all users were willing to allow sufficient equipment in their homes or make adaptations to their lifestyle, adaptions that would accommodate the requirements of safe working conditions. Here, one participant discusses the issue: ‘*I have several users who have very low beds and refuse to buy something new’.* Moreover, the design of the environment in users` homes makes it difficult to use the appropriate equipment and maintain ergonomic principles: *‘I have to stand outside the room and bend my back over him to be able to assist him on the toilet …. And after, I feel pain in my back’.*

Several male participants reported challenges regarding following and maintaining ergonomic guidelines because of their height and body size: *‘I am very tall … .ehh …. Yes, so one of the most strenuous things I do...it is in the kitchen... with low or just regular kitchen benches … so then I get an awkward bend and strenuous work postures on the neck, back, and shoulders...and I get stiff or feel some pain in the musculature‘.*

#### The perceived responsibility in complying with company safety policies and practices

The participants highlighted that each worker is responsible for engaging in the appraisal and facilitation of a healthy workplace: ‘*You also have your own responsibility to report strain or unhealthy conditions early enough and take care of your own health’.*

The participants also expressed that it is their responsibility to report the need for ergonomic facilitation, to seek help from other colleagues when demanding tasks arise, to help others in such situations, to switch work tasks that exceed their physical capabilities, and to report on demanding users and whatever health risks may present themselves in their homes. On the other hand, since the workplaces for workers are people’s homes, and since each one is designed and shaped differently, the participants experienced that their working conditions changed from user to user. Accordingly, several experienced receiving too much individual responsibility when it came to creating healthy working conditions in the workplace. Several challenges were reported regarding the issue. When visiting users’ homes, participants assess the situation, set personal boundaries, and make a personal judgement regarding whether they are willing to work in the environment. This leads to considerably varied work situations, where each individual’s perceptions have a major influence on how they judge their work experience: ‘*One problem is different perceptions among peers …. We are different … so it is a bit challenging’.*

Some participants reported they always performed the work tasks they received and who even took on extra tasks even if they were unhealthy. They experienced more physical strain and unhealthy body positions when compared to other workers who were more careful and set stricter limits to protect their bodies from harm. Two participants discussed this issue. The first worker stated a desire to avoid becoming hurt: *‘I refuse and never lift at work … after all, we will have our backs for many years and we have to take care of ourselves’.* On the other hand, the other participant made this declaration: *‘ … even though we should not lift, I always lift at work’.*

When workers refused to do specific work tasks to take care of their health, they often did so at the expense of their colleagues’ health because another worker would eventually have to perform the rejected task. One participant highlighted the troublesome nature of this predicament: *‘I am very strict and require equipment to be available in users’ homes.. otherwise I’m not going there’.* Another participant described a different view: ‘*I do not always have the opportunity to perform the work ergonomically correctly … and then I do the tasks at the expense of my health because … the job has to be done’.*

The participants reported that not all workers dared to express their needs by reporting unfortunate conditions they would like to be changed: ‘*Sometimes there are discussions about whether their needs to be two workers in a user’s home or not … but some need more help from colleagues than others’*. Another participant said*: ‘If you need help ...then you get it...but you have to ask for it...but I think not all dare to ask’.*

Participants reported that feelings of responsibility for the users influenced their decision making, causing them to push the limits. Several reported that they felt the job had to be done and didn’t have the conscience to say no to extra tasks. One participant described her feelings regarding this dilemma in these words: *‘But I feel it’s my responsibility to do the task … the users need to get out of their bed … and I can’t just go’.* Another participant said: *‘It’s really my fault that I worked with pain...because I didn’t prioritise my health and pushed myself’.*

### Organisational conditions

The participants addressed shift work, time pressure and sick leave as organisational factors that negatively impact their health. Performing tasks in accordance with their professional skills and identity promoted health.

#### Shift work, time pressure and staffing challenges as health-impairing factors

The disturbed sleep patterns and reduced sleep quality that shift work causes was reported to lead workers to feel unrefreshed and poorly rested: *‘To go from an afternoon shift to a morning shift the next day, affects my sleep in a negative way... I’m not getting enough sleep...it’s not so good for my health’.*

The participants reported that the organisation of their work tasks and additional increasing demands created time pressure and hectic workdays. Unexpected incidents such as transferring over new geographic areas, performing tasks that take more time than has been allocated, and taking care of users sent home from the hospital at an early stage of recovery created time pressure and were perceived as detrimental to the health of workers.

Regarding the health consequences of being exposed to time pressure, the participants reported mental and emotional strain, exhaustion, and burnout: *‘The time pressure is the worst thing about the job...it is quite catastrophic at times’.* The side-effects of time pressure were skipping lunch, not going to the toilet, experiences of dizziness and nausea, blood pressure problems, seizures and palpitations, headaches, tension, and strain in the neck and shoulders. Furthermore, the participants felt bad for not doing enough for the users and said that there is little time to meet their psychological and social needs: *‘I get upset because I can’t spend more time talking to the users’.*

Staffing challenges were reported to occur, especially when colleagues were on sick leave. Sick leave was often needed due to the combined psychological and physical demands of their work conditions: *‘I ended up on sick leave...because we have too much to do … and we also use our body all the time.... I am constantly pushing and pushing myself.... I was exhausted and really needed a break … then it said stop...and I got burned out’.*

Excessive use of sick leave created staffing challenges and added more pressure and stress as it required the remaining workers to perform extra tasks besides those detailed in their original work schedules and to take on extra responsibilities to fill care gaps and meet the needs of users: *‘I often feel pressured to take on extra work tasks...it’s difficult to say no … I am feeling very tired … it is demanding to be at work’.* The challenge was reported by all participants regardless of their professional background.

#### Facilitating professional identity

The participants reported that they were assigned tasks they perceived as appropriate to their professional competence and identity. This was very much appreciated and considered as a positive influence on their health: *‘It is important for me to use my education and skills as a nurse...it is exciting if I get tasks that are a bit more complex...like sterile procedures … intravenous...I like that’.*

Being able to use one’s professional competence at work was reported as being both meaningful and crucial to job satisfaction. Participants felt that it strengthened their professional identity as nurses, occupational therapists, and home health aides. Furthermore, participating in specific work tasks relating to one’s education also positively influences mental health. Such tasks contributed to workers’ independence, sense of confidence, and self-efficacy. The participants experienced that their workplace facilitated their professional identity and growth when it came to providing important sources of specialist knowledge in the form of special training and further education. Participants viewed this as another factor positively influencing their health.

## Discussion

HCWs are providers of important health services and social care. The number of people in need of home services is expected to increase in the future, without corresponding access to labour and skills. HCWs experience the work as physically demanding, stressful and exhausting, have high sickness absence that is often attributed to musculoskeletal disorders, and a high probability of disability pension [1, 13–15]. Therefore, it is necessary to put in place effective organisational measures to reduce poor health, sickness absence, and disability among HCWs to be able to provide good services to the population in the future. This qualitative study contributes insights on the connection between HCWs experiences of work conditions and their perceived health in a Norwegian context.

### Work-related relationships

The results demonstrate that relationships with colleagues, managers, users, and families contribute to good health among the HCWs. These findings are in line with some previous international studies, demonstrating that close and meaningful relationships with users and their families have positive mental health effects [30–40]. In contrast, findings from several studies have highlighted that HCWs can experience little to no contact with colleagues and can feel lonely at work [32–34, 39–42]. These conditions can contribute to workers becoming further isolated from conventional personal support systems and can also lead to the build-up of emotional stress [34, 42].

However, the findings in this study show that there is a flip side to having a meaningful and close relationship with the users. Emotional strain and stressors were experienced as consequences of becoming too emotionally attached to users, especially users who suffer due to severe health conditions or died. Similar findings have been reported in several studies, [30, 34, 36, 38, 40, 43–45], and the perceived risk of increased psychosocial strain associated with relationships has also been researched [35]. Moreover, our results show that constructive relationships sometimes can be difficult to establish and can contribute to poor health. Findings from both Norwegian [46–48] and international studies [30, 34, 36, 38, 42–44, 49–53] have reported experiences of threats and violence at users homes as a contributor to HCWs poor mental health stress disorders, and emotional exhaustion [51]. The psychological strain experienced by HCWs can be intense because these workers largely work alone [49–51]. Health and safety issues present in the home care services may be difficult to resolve in the manner they are solved in other health services. For example, in hospitals or nursing homes, employees have better access to human resources or security personnel than HCWs [51]. Adding more security structures can restrict users and institutionalise their homes.

### An unpredictable workplace

The findings from the present study offer an in-depth understanding of how unhealthy work conditions occur as a consequence of the unpredictable work conditions they encounter, not because of a lack of focus or lack of measures enacted by the employer and workplace managers. The participants enlisted environmental health hazards as challenging driving conditions and fall hazards during winter, unhygienic environment and tobacco smoke at some users` homes. Physically straining tasks conducted in a non-ergonomic work situation, including unhealthy body positions, were common. These findings are supported by other scientific literature [8, 10, 32, 34, 36, 38, 39, 42, 52–57].

Due to the decentralisation reforms that came into effect during the mid-1980s, an increased number of people began receiving treatment and follow-up at home [2]. As a result, involvement and empowerment among the users have been strengthened [58]. Workplace and the HCWs working conditions are tailored in a space where formal home care services systems, regarded as the concept ‘gesellschaft’, interact with users’ life-worlds, considered as ‘gemeinschaft’ [59]. In order to enhance users’ life-worlds as expressed in the form of values, roles, and habits, HCWs sometimes must work in conditions that are not beneficial for their health. For example, not all users showed support or allowed changes to be made in their homes that would create an ergonomic workplace. Users were also not willing to stop smoking tobacco while receiving care. Exposure to second-hand smoke has also been documented in previous studies [35, 36, 52–54, 57, 60].

Moreover, our findings suggest the participants perceived a strong responsibility for complying with institution safety policies and practices. Yes, the employer and the leadership were reported to have a positive and active approach to creating healthy work conditions. The employer promoted ergonomic work practices such as ergonomic body mechanics when mobilising and handling clients and using safe patient handling equipment. However, the participants perceived it as it was to the high degree up to them as individuals to assess and communicate what they perceived as needed in different homes. This requires a high level of assessment competence, it can appear time-consuming, and it opens for individual perceptions, which are not necessarily strengths when creating healthy work conditions. This, in turn, can create major differences in terms of how employees experience their work conditions and how the work conditions affect the health of individual workers. For example, some employees experienced increasingly impaired health compared to others who were more careful and set stricter limits to protect themselves. It has been documented that some workers, to a greater extent than others, set aside their health and safety needs to accomplish work tasks [54, 61].

HCWs experiences depicted in the present study are important to understand how they resolve health and safety issues in an unpredictable workplace. However, to fully understand HCWs occupational safety and health issues and responsibilities, it is important that institution managers and leaders and occupational safety and health policymakers and personnel at the institution are interviewed to understand their perspectives of the situation. The leadership have the ultimate legal responsibility to provide their employees with safe working conditions.

### Organisational conditions

The findings of this study also show that the distribution of work tasks according to employees’ professional skills and identity enhances employees` growth and health, and providing opportunities for professional development strengthens employees’ sense of autonomy, which is in line with previous findings [55].

Shift-work and work task organisation and increased demands create time pressure and stress. It is well established that time pressure is experienced as one of the most strenuous factors for HCWs [9, 13–15, 17, 32, 33, 37, 38, 62] and that it may create negative emotions such as frustration and stress [15, 33, 37, 62].

Staffing challenges resulting from colleagues on sick leave were reported to make HCWs workplace even more unpredictable. This finding is in line with the results from the qualitative study conducted by Andersen and Westgaard [13]. Home care units had challenges to replace absent employees. It created a need for the re-allocation of resources, which again added more pressure and stress for the remaining employees who were still at work. All participants reported that they often took over some of the sick person’s tasks. Sick leave is an earned, hard-won benefit to which the workers have the right. Also, workers cannot go sick in users’ homes because this could result in a serious safety or health situation. Fundamentally, the safety and health of workers and consumers go hand-in-hand. Given that the home care services have high sick leave rates, and if they fall short of coping with the staffing challenges it creates, it can pose a substantial challenge for HCWs health, resulting in even higher sick leave.

In line with previous research, participants in the present study reported medical conditions such as muscular and pain disorders, especially those associated with the neck, shoulders, feet, and back [9, 10, 13, 15, 16, 34–36, 40, 42, 43, 54, 56, 57, 60, 61, 63–65]. This study contributes to understanding unpredictable work situations in users’ homes that can lead to health-impairing situations, even when workers and managers strive to create a health-promoting workplace. The findings suggest the context is perceived to require a high degree of individual responsibility for complying with institution safety policies and practices. Both competence and subjective approaches can create obstacles for HCWs while taking that responsibility, despite best intentions. Still, even if workers live up to the challenge, they may be prevented from creating healthier work conditions because they cannot always control their working environment. Staffing challenges were common due to high sick leave, which generates more tasks for those who are at work. It is an additional burden. Addressing this unfortunate interplay of factors can be challenging as research suggests that stakeholders at different institutions have different perceptions of the situation [15]. They don’t share the same working environment after all. However, more insight and knowledge is a contribution to all stakeholders.

### Implications for theory, practice and research

The findings reveal the need to develop new frameworks on how employees` health can be ensured and promoted in an environment that lies on the crossing point between users` homes and employees` workplaces without institutionalising users` homes. It also informs the practice field about the need to address the challenge. Measures might be needed to address the following crucial questions: What is the right balance between users` autonomy and empowerment at their homes and the need for health-promoting work conditions for HCWs? How can HCWs individual responsibility for the promotion of healthy work conditions be minimised? How to avoid additional strain for those at work when their colleagues take sick leave?

The present study offers HCWs perspectives on how working conditions influence their safety, health, and wellbeing. However, home care services are institutions with many stakeholders. They may have different needs, interests and prerequisites. Future research should bring in additional perspectives by exploring attitudes and experiences of home care agency managers/directors, union representatives, local and government authorities responsible for the occupational safety and health administration. It is also crucial that stakeholders get the opportunity to address the question of how challenging working conditions can be minimised and eliminated in an unpredictable working environment while users` autonomy is maintained.

## Methodological considerations and limitations

The age of the participants is relatively low, and the experiences can be perceived as homogeneous. Several participants with high seniority may have added more depth to the findings. On the other hand, the sample in the present study reflects the HCWs age in the home care units we included.

Following Malterud et al.’s model of information power, [66] the adequacy of the sample size was continuously evaluated during data collection. Information power was ensured by participants who possessed broad experience with home care services, strong dialogue-based interviews, and performance of appropriate thematic analysis [66]. In addition, we also recorded a saturation and believe the sample provides enough information power to answer the research question and generate new knowledge. Hence, studying a phenomenon in-depth and in detail does not require large study samples [67]. We also recruited from more than one study site to strengthen the findings’ external validity and made them more easily transferable to practice. On the other side, additional details could have emerged if more workers had been interviewed (e.g., themes related to bloodborne pathogen exposures, work hazards from medical equipment).

Furthermore, the participants had different occupations (four nurses, one occupational therapist, three home aides). They had different qualifications and responsibilities. Therefore, it is unlikely that they narrate from entirely similar perspectives. Nurses and occupational therapists typically perform skilled tasks that home health aides may not be qualified for undertaking. Similarly, home health aides may perform strenuous personal care and homemaking tasks to support home care consumers in their activities of daily living. For example, how musculoskeletal disorders and stress are experienced may relate to professional responsibilities and work tasks. On the other side, the differences in their perspectives contribute to a comprehensive and complementary grasp of the phenomenon.

## Conclusions

This study suggests that unpredictable working conditions at users’ homes can adversely affect the safety, health, and wellbeing of HCWs, despite the strong focus on ergonomics and health promotion at an organisational level. HCWs meaningful relationships with users, their families, colleagues and performing tasks in accordance with their professional skills identity promote health. The interplay between the unpredictable conditions HCWs meet at users` homes, HCWs’ perceived high level of individual responsibility for complying with company safety policies and practices, and staffing challenges due to sickness-related absences in the workplace create work conditions that negatively influence HCWs health.

Both theory and practice should be reexamined to address the challenge without compromising users` autonomy and empowerment.

## Data Availability

The datasets used and/or analysed during the current study are available from the corresponding author on reasonable request.

## Abbreviations

HCWs: Home Care Workers
COREQ: Consolidated criteria for Reporting Qualitative research
